# DHHC5 regulates lacteal function and intestinal lipid absorption by maintaining VEGFR2 localization in lipid rafts

**DOI:** 10.1093/lifemeta/loaf014

**Published:** 2025-04-10

**Authors:** Yin-Yue Zhao, Yi-Fan Li, Jian-Wei Hao, Ning Zhao, Xiao-Ting Men, Xiao-Yu Bai, Rui Tai, Hao-Bin Ye, Xing-Rong Du, Hui-Ling Guo, Juan Wang, Hong-Jie Qian, Tong-Jin Zhao

**Affiliations:** State Key Laboratory of Genetic Engineering, Shanghai Key Laboratory of Metabolic Remodeling and Health, Institute of Metabolism and Integrative Biology, Zhongshan Hospital, Fudan University, Shanghai 200438, China; State Key Laboratory of Genetic Engineering, Shanghai Key Laboratory of Metabolic Remodeling and Health, Institute of Metabolism and Integrative Biology, Zhongshan Hospital, Fudan University, Shanghai 200438, China; State Key Laboratory of Cellular Stress Biology, School of Life Sciences, Xiamen University, Xiamen, Fujian 361102, China; State Key Laboratory of Cellular Stress Biology, School of Life Sciences, Xiamen University, Xiamen, Fujian 361102, China; State Key Laboratory of Cellular Stress Biology, School of Life Sciences, Xiamen University, Xiamen, Fujian 361102, China; State Key Laboratory of Genetic Engineering, Shanghai Key Laboratory of Metabolic Remodeling and Health, Institute of Metabolism and Integrative Biology, Zhongshan Hospital, Fudan University, Shanghai 200438, China; State Key Laboratory of Genetic Engineering, Shanghai Key Laboratory of Metabolic Remodeling and Health, Institute of Metabolism and Integrative Biology, Zhongshan Hospital, Fudan University, Shanghai 200438, China; State Key Laboratory of Genetic Engineering, Shanghai Key Laboratory of Metabolic Remodeling and Health, Institute of Metabolism and Integrative Biology, Zhongshan Hospital, Fudan University, Shanghai 200438, China; State Key Laboratory of Genetic Engineering, Shanghai Key Laboratory of Metabolic Remodeling and Health, Institute of Metabolism and Integrative Biology, Zhongshan Hospital, Fudan University, Shanghai 200438, China; State Key Laboratory of Genetic Engineering, Shanghai Key Laboratory of Metabolic Remodeling and Health, Institute of Metabolism and Integrative Biology, Zhongshan Hospital, Fudan University, Shanghai 200438, China; State Key Laboratory of Cellular Stress Biology, School of Life Sciences, Xiamen University, Xiamen, Fujian 361102, China; State Key Laboratory of Genetic Engineering, Shanghai Key Laboratory of Metabolic Remodeling and Health, Institute of Metabolism and Integrative Biology, Zhongshan Hospital, Fudan University, Shanghai 200438, China; Drug Clinical Trial Center, Shanghai Xuhui Central Hospital/Xuhui Hospital, Fudan University; Shanghai Engineering Research Center of Phase I Clinical Research and Quality Consistency Evaluation for Drugs, Shanghai 200237, China; State Key Laboratory of Genetic Engineering, Shanghai Key Laboratory of Metabolic Remodeling and Health, Institute of Metabolism and Integrative Biology, Zhongshan Hospital, Fudan University, Shanghai 200438, China; Innovation Center of Basic Research for Metabolic-Associated Fatty Liver Disease, Ministry of Education of China; Tianjian Laboratory of Advanced Biomedical Sciences, Academy of Medical Sciences, Zhengzhou University, Zhengzhou, Henan 450001, China

**Keywords:** DHHC5, intestinal lipid absorption, lacteals, palmitoylation, VEGFR2

## Abstract

The intestinal lymphatic system is essential for lipid absorption, yet its regulatory mechanisms remain poorly understood. Here, we identify DHHC5, an Asp-His-His-Cys (DHHC) motif-containing palmitoyl acyltransferase, as a critical regulator of intestinal lymphatic integrity and lipid uptake. Whole-body inducible *Dhhc5* knockout (*Dhhc5-IKO*) mice were resistant to diet-induced obesity and exhibited impaired intestinal lipid absorption due to lymphatic dysfunction. Similar defects were observed upon specific knockout of *DHHC5* in lymphatic endothelial cells (LECs), underscoring its cell-autonomous role. Mechanistically, DHHC5 facilitates vascular endothelial growth factor receptor 2 (VEGFR2) signaling by promoting its lipid raft localization in LECs. We further identified CRYBG1, an actin-binding protein, as the substrate of DHHC5. CRYBG1 interacts with VEGFR2, and its palmitoylation is required for the lipid raft localization of VEGFR2. These findings reveal a DHHC5–CRYBG1–VEGFR2 axis that governs intestinal lymphatic function and lipid absorption, providing new insights into the regulation of dietary lipid metabolism.

## Introduction

Dietary lipid absorption is a critical regulatory step in lipid metabolism, with the small intestine playing a central role in this process [1]. Excessive intestinal lipid absorption contributes to obesity and related metabolic syndromes, while impaired fat absorption can lead to developmental delays due to deficiencies in essential fatty acids and fat-soluble vitamins [2–4]. Lacteals, specialized lymphatic capillaries that are located in the center of intestinal villi [5], are essential for transporting chylomicrons released from enterocytes [6–9]. Dysfunction of lacteals is associated with significant pathologies. For example, in patients with lymphangiectasia, lacteals become dilated or obstructed, resulting in impaired fat absorption, malnutrition, diarrhea, and weight loss [10]. Additionally, altered lacteal function has been linked to obesity and type 2 diabetes [11]. Despite their physiological and clinical importance, the mechanisms regulating lacteal function remain poorly understood.

Recent studies have begun to unravel the complex regulatory mechanisms governing lacteal development and function, with a particular focus on vascular endothelial growth factor receptor 2 (VEGFR2) [12]. VEGF-A-mediated VEGFR2 activation is essential for lymphangiogenesis during embryonic development [13, 14]. In addition, VEGF-A-VEGFR2 signaling regulates the formation of “button-like” cell junctions in lymphatic endothelial cells (LECs), a structural feature critical for the entry of chylomicrons into lacteals [12, 15]. However, the mechanisms regulating VEGFR2 activity and localization in LECs remain poorly understood.

Protein S-palmitoylation, the reversible addition of fatty acyl chains to proteins, is a key posttranslational lipid modification [16]. This process not only stabilizes the membrane localization of raft-associated proteins but also facilitates their functional interactions within these specialized domains [17–19]. Our previous work demonstrated that the Asp-His-His-Cys (DHHC) motif-containing palmitoyl acyltransferase DHHC5 regulates fatty acid uptake in adipose tissue by dynamically palmitoylating CD36 [20, 21]. Beyond adipose tissue, DHHC5 has been implicated in diverse physiological processes, including cardiac function [22] and neuronal activities [23–25]. However, the role of DHHC5 in whole-body metabolism and other tissues remains largely unexplored.

To address the question, we generated whole-body inducible *Dhhc5* knockout (*Dhhc5-IKO*) mice. These mice exhibited impaired intestinal lipid uptake, which we attributed to defects in the lymphatic system. Mechanistically, we demonstrate that DHHC5 palmitoylates CRYBG1, an actin-binding protein, to promote the lipid raft association of VEGFR2, thereby regulating VEGFR2 signaling in LECs. Our findings reveal a critical role for protein palmitoylation in controlling the function of the intestinal lymphatic system and highlight the DHHC5–CRYBG1–VEGFR2 axis as a key regulatory mechanism.

## Results

### Inducible knockout of *Dhhc5* in adult mice prevents diet-induced obesity

**Figure 1:**
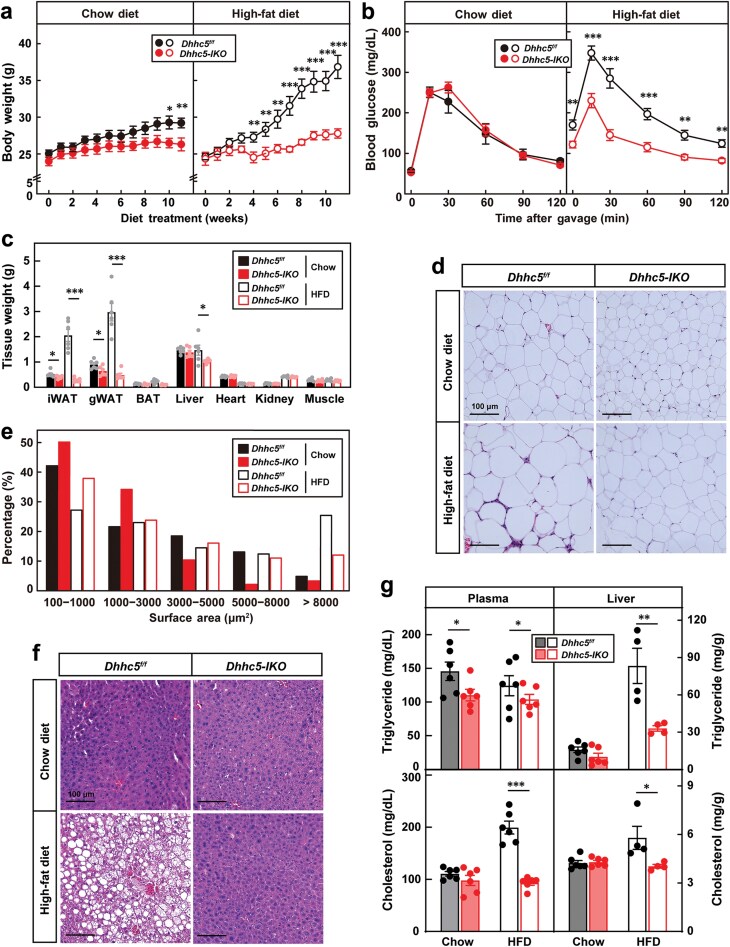
Knockout of *Dhhc5* in adult mice protects against diet-induced obesity. On Week 8, control (*Dhhc5*^*f/f*^) and *Rosa-Cre*^*ERT*^; *Dhhc5*^*f/f*^ (*Dhhc5-IKO*) male mice were gavaged with tamoxifen (20 mg/kg) for five times. Starting from Week 9, mice were subjected to chow die or HFD feeding for 10 weeks. (a) Body weight monitored every week. Each value represents mean ± SEM of eight mice. (b) Glucose tolerance test. After 8 weeks of feeding, mice were fasted for 16 h (from 5 p.m. to 9 a.m.), and subjected to glucose tolerance test, 2 mg/g glucose for chow diet-fed mice and 1 mg/g for HFD-fed mice. Blood glucose was monitored at indicated time. Each value represents mean ± SEM of eight mice. (c) Tissue weights recorded after sacrifice of the mice. Each value represents mean ± SEM of eight mice. (d–f) H&E analysis of gWAT and liver. After dissection, gWAT (d) and liver (f) were subjected to H&E analysis. Scale bar, 100 μm. The sizes of adipocytes were quantified in (e). (g) Plasma or liver triglyceride and cholesterol were measured using commercial kits. Each value represents mean ± SEM of five mice. Asterisks (*) denote level of statistical significance (Student’s *t*-test) between *Dhhc5*^*f/f*^ and *Dhhc5-IKO* mice. *P < 0.05; **P < 0.01; ***P < 0.001. See also Supplementary Fig. S1.

To investigate the role of DHHC5 in whole-body metabolism, we generated *Dhhc5-IKO* mice by crossing *Dhhc5*^*f/f*^ mice [20] with *Gt(ROSA)26Sor*^*tm1(cre/ERT2)Tyj*^ mice [26]. At 8 weeks of age, mice were treated with tamoxifen to induce *Dhhc5* deletion (Supplementary Fig. S1a). *Dhhc5-IKO* and control mice were then fed either a chow diet or a high-fat diet (HFD). On a chow diet, *Dhhc5-IKO* mice exhibited a modest reduction in body weight after 10 weeks (Fig. 1a). Strikingly, *Dhhc5-IKO* mice were resistant to HFD-induced weight gain (Fig. 1a). While glucose tolerance was similar between chow diet-fed groups, HFD-fed *Dhhc5-IKO* mice displayed improved glucose clearance (Fig. 1b).

At the end of the feeding period, tissues were collected for analysis. Consistent with their reduced body weight, *Dhhc5-IKO* mice had lower adipose tissue mass compared to the controls, a difference that was more pronounced under HFD conditions. Liver weight was also reduced in *Dhhc5-IKO* mice (Fig. 1c). Histological analysis revealed that white adipocytes in HFD-fed *Dhhc5-IKO* mice were smaller than those in the controls (Fig. 1d and e; Supplementary Fig. S1b). Additionally, HFD-induced whitening of brown adipose tissue was attenuated in *Dhhc5-IKO* mice (Supplementary Fig. S1b). *Dhhc5* knockout also significantly reduced HFD-induced hepatic steatosis, as well as plasma and liver triglyceride and cholesterol levels (Fig. 1f and g).

### Loss of DHHC5 decreases intestinal lipid absorption

To explore the mechanism by which DHHC5 loss prevents diet-induced obesity, we first assessed energy balance using metabolic cage analysis. No differences were observed between *Dhhc5*^*f/f*^ and *Dhhc5-IKO* mice in food intake, energy expenditure, or physical activity (Supplementary Fig. S2).

**Figure 2:**
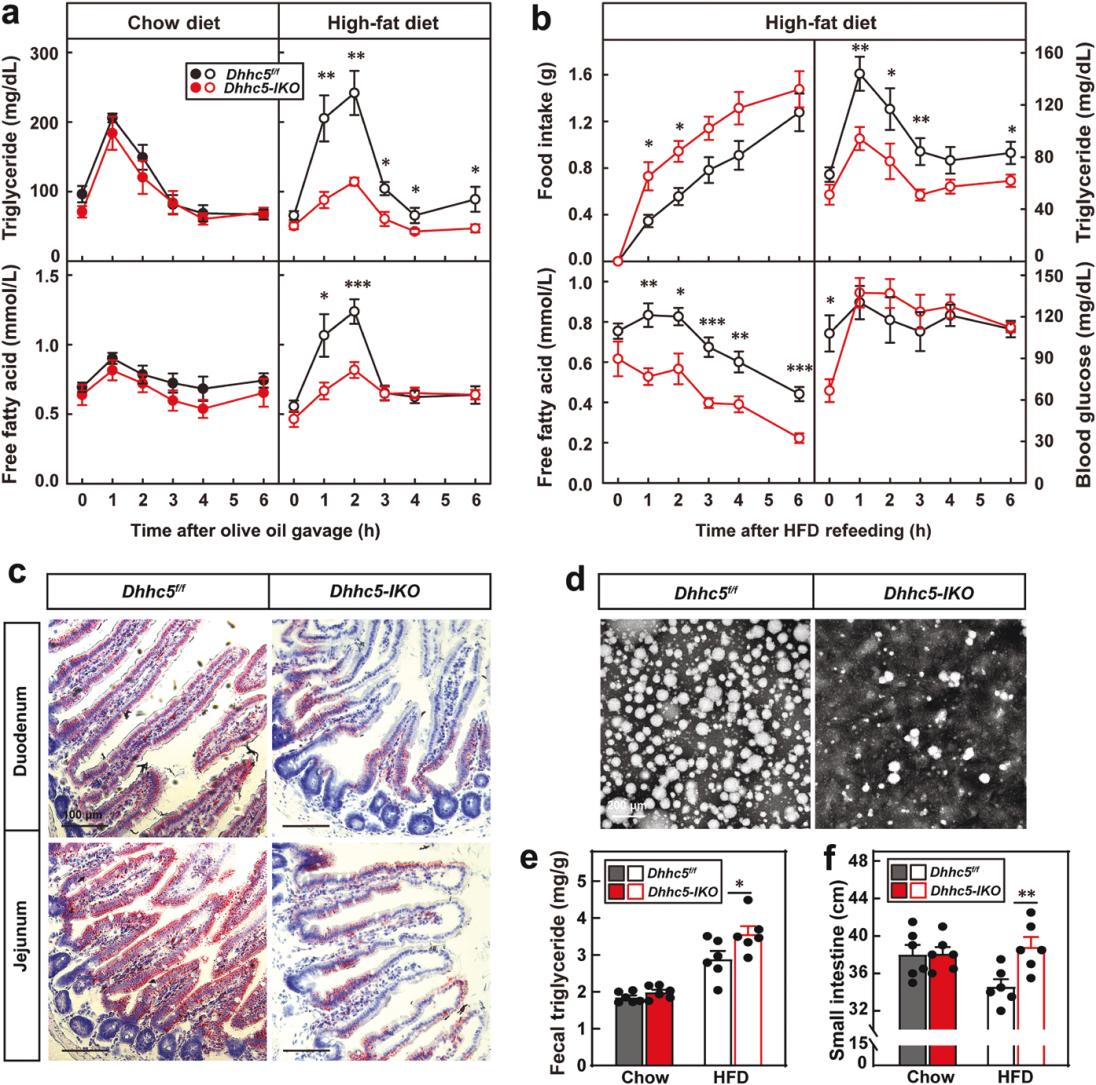
*Dhhc5-IKO* mice have defects in intestinal lipid absorption. (a) Plasma levels of triglyceride and free fatty acids. After 8 weeks of chow diet or HFD feeding, *Dhhc5*^*f/f*^ and *Dhhc5-IKO* male mice were briefly fasted for 4 h, and gavaged with olive oil at 6.5 μL/g body weight. Blood samples were collected from the tail vein at indicated time for measurement of plasma levels of triglyceride and free fatty acids. (b) Levels of food intake, blood glucose, plasma triglyceride, and free fatty acids. After 10 weeks of HFD feeding, *Dhhc5*^*f/f*^ and *Dhhc5-IKO* male mice were fasted for 16 h in single cages, and then refed with HFD for 6 h. Food intake, blood glucose, plasma triglyceride and free fatty acids were measured every hour. (c) Oil Red O staining of small intestines. HFD-fed *Dhhc5*^*f/f*^ and *Dhhc5-IKO* mice (16-week-old, male) were fasted for 4 h and then gavaged with olive oil for 2 h. Small intestines were collected for Oil Red O staining. Scale bar, 100 μm. (d) Observation of chylomicrons isolated from blood using electronic microscopy. HFD-fed *Dhhc5*^*f/f*^ and *Dhhc5-IKO* mice (16-week-old, male) were fasted for 16 h and then refed with HFD for 1 h. Chylomicrons were isolated from blood and observed by electronic microscopy. Scale bar, 200 nm. (e) Fecal triglyceride. After 12 weeks of chow or HFD feeding, *Dhhc5*^*f/f*^ and *Dhhc5-IKO* male mice were singly housed for 2 days. Feces were collected for measurement of triglyceride. (f) Length of small intestine. At the end of feeding, chow diet or HFD-fed *Dhhc5*^*f/f*^ and *Dhhc5-IKO* male mice were sacrificed and the length of the small intestine was measured. Each value represents mean ± SEM of six mice. Asterisks (*) denote the level of statistical significance (Student’s *t*-test) between *Dhhc5*^*f/f*^ and *Dhhc5-IKO* mice. *P < 0.05; **P < 0.01; ***P < 0.001. See also Supplementary Fig. S2.

We next investigated whether *Dhhc5-IKO* mice exhibited defects in intestinal lipid absorption. Following oral gavage of olive oil, chow diet-fed *Dhhc5-IKO* and control mice showed similar plasma triglyceride and free fatty acid levels. In contrast, HFD-fed *Dhhc5-IKO* mice displayed significantly lower plasma levels of these lipids (Fig. 2a), indicating impaired intestinal lipid absorption.

To further confirm this finding, we subjected mice to fasting and refeeding. Despite consuming more food, HFD-fed *Dhhc5-IKO* mice showed markedly reduced plasma triglyceride and free fatty acid levels compared to the controls, while glucose uptake remained unaffected (Fig. 2b). Histological analysis of the duodenum and jejunum 2 h after refeeding revealed significantly lighter Oil Red O staining in *Dhhc5-IKO* mice (Fig. 2c), further supporting defective lipid absorption.

To validate these results, we isolated chylomicrons from HFD-fed mice. *Dhhc5-IKO* mice exhibited lower plasma chylomicron levels and smaller chylomicron sizes compared to the controls (Fig. 2d). Consistent with impaired lipid absorption, HFD-fed *Dhhc5-IKO* mice had higher fecal triglyceride content (Fig. 2e) and significantly longer small intestines (Fig. 2f).

Together, these findings demonstrate that whole-body knockout of *Dhhc5* leads to decreased intestinal lipid absorption in HFD-fed mice.

### Knockout of *Dhhc5* in LECs decreases intestinal lipid absorption

To identify the target tissue through which DHHC5 regulates intestinal lipid absorption, we considered multiple organs involved in this process: the pancreas (digestive enzyme production), liver (bile acid production), and small intestine (enterocytes for lipid uptake and the lymphatic system for lipid transport). We generated tissue-specific *Dhhc5* knockout mice by crossing *Dhhc5*^*f/f*^ mice with *Pdx1-Cre* (pancreas), *Albumin-Cre* (liver), or *Villin-Cre* (enterocytes) mice. However, none of these models recapitulated the phenotypes observed in *Dhhc5-IKO* mice (Supplementary Fig. S3). Specifically, pancreas-, liver-, and enterocyte-specific *Dhhc5* knockout mice showed no differences in HFD-induced weight gain or intestinal lipid absorption compared to the controls (Supplementary Fig. S3). These results suggest that DHHC5 does not regulate intestinal lipid absorption through the pancreas, liver, or enterocytes.

**Figure 3:**
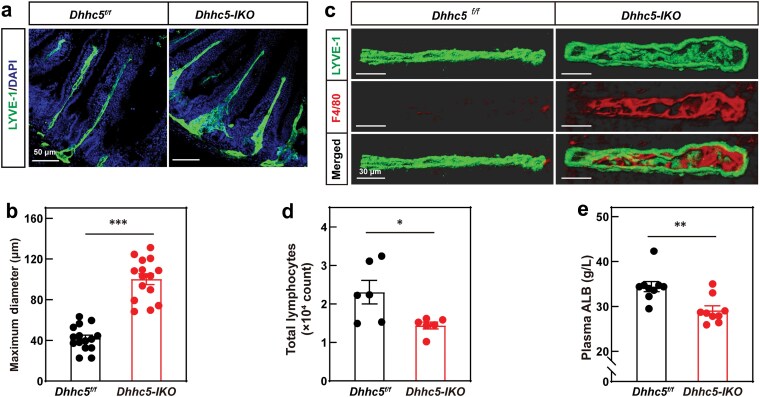
*Dhhc5-IKO* mice have defects in intestinal lymphatic system. (a–c) Histological analysis and immunostaining of the intestinal lymphatic system. *Dhhc5*^*f/f*^ and *Dhhc5-IKO* mice were fed on an HFD for 12 weeks. After dissection, jejuna from HFD-fed *Dhhc5*^*f/f*^ and *Dhhc5-IKO* mice were subjected to whole-mount immunofluorescent staining to observe lacteals. Scale bar, 50 μm (a). The maximum diameter of lacteals was quantified in (b). Each data point represents a single lacteal, with three mice per group. Data are expressed as the mean ± SEM. Images of the jejunum stained with LYVE-1 and F4/80 antibodies were reconstructed by Imaris. Scale bar, 30 μm (c). (d and e) Flow cytometry analysis of lymphocytes and plasma albumin measurement. *Dhhc5*^*f/f*^ and *Dhhc5-IKO* mice were fed on an HFD for 10 weeks. Blood samples were collected from the orbital vein and subjected to flow cytometry analysis of lymphocytes (d) and plasma albumin measurement (e). Each value represents mean ± SEM of six mice. Asterisks (*) denote level of statistical significance (Student’s *t*-test) between *Dhhc5*^*f/f*^ and *Dhhc5-IKO* mice. *P < 0.05; **P < 0.01; ***P < 0.001.

Given the critical role of the intestinal lymphatic system in lipid absorption and transport, we next investigated whether *Dhhc5-IKO* mice exhibited defects in this system. Histological analysis revealed that lymphatic vessels at the base of intestinal villi were significantly broader in *Dhhc5-IKO* mice (Fig. 3a and b). Immunostaining showed increased macrophage (F4/80) signals in the lacteals of *Dhhc5-IKO* mice, suggesting potential blockage and inflammation (Fig. 3c). Consistent with these findings, *Dhhc5-IKO* mice had reduced blood lymphocyte counts (Fig. 3d; Supplementary Fig. S4) and lower plasma albumin levels (Fig. 3e). These phenotypes closely resemble those of intestinal lymphangiectasia, further implicating lymphatic dysfunction in *Dhhc5-IKO* mice.

**Figure 4:**
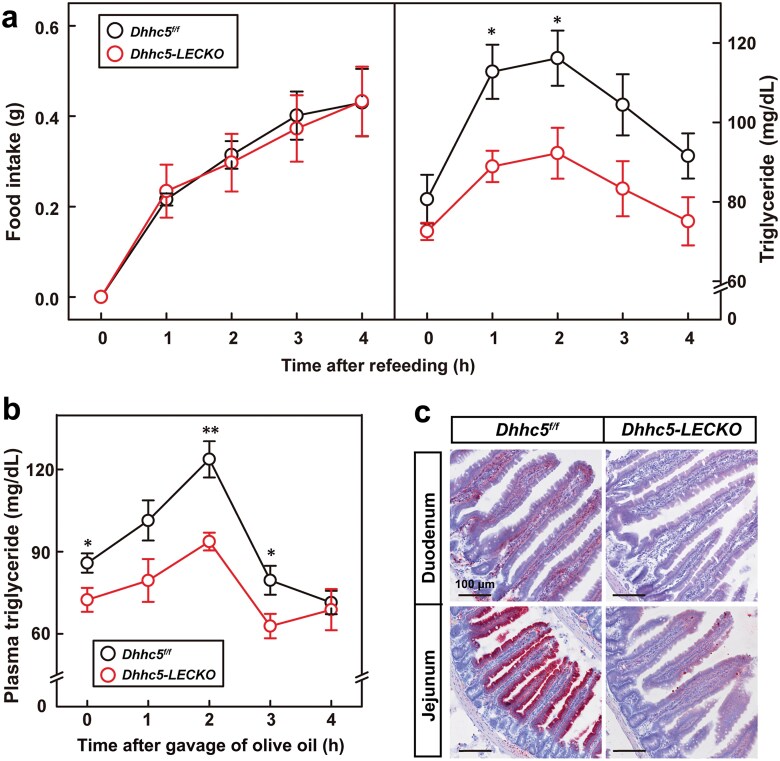
HFD-fed *Dhhc5-LECKO* mice have defect in intestinal lipid absorption. On Week 8, *Dhhc5*^*f/f*^ and *Dhhc5-LECKO* male mice were gavaged with tamoxifen (20 mg/kg) for five times. Starting from Week 9, mice were subjected to HFD feeding for 11 weeks. (a) Food intake and plasma triglyceride. *Dhhc5*^*f/f*^ and *Dhhc5-LECKO* mice were fed with HFD for 13 weeks and were gavaged with tamoxifen for once. Mice were fasted for 16 h in single cages, and then refed with HFD for 4 h. Food intake and plasma triglyceride were measured every hour. Each value represents mean ± SEM of seven mice. (b) Plasma levels of triglyceride after oral gavage of olive oil. *Dhhc5*^*f/f*^ and *Dhhc5-LECKO* mice were fed with HFD for 15 weeks and were gavaged with tamoxifen for once. One week later, mice were briefly fasted for 4 h, and gavaged with olive oil at 6.5 μL/g body weight. Blood samples were collected from the tail vein at indicated time for measurement of plasma levels of triglyceride. Each value represents mean ± SEM of six mice. (c) Oil Red O staining of intestinal tissues. *Dhhc5*^*f/f*^ and *Dhhc5-LECKO* mice were fed with HFD for 17 weeks and were gavaged with tamoxifen for once. One week later, mice were briefly fasted for 4 h and then gavaged with olive oil for 2 h. Small intestines were collected for Oil Red O staining. Scale bar, 30 μm. Asterisks (*) denote level of statistical significance (Student’s *t*-test) between *Dhhc5*^*f/f*^ and *Dhhc5-LECKO* mice. *P < 0.05; **P < 0.01.

To confirm the role of DHHC5 in LECs, we generated LEC-specific *Dhhc5* knockout mice (*Dhhc5-LECKO*) by crossing *Dhhc5*^*f/f*^ mice with *Prox1-Cre*^*ERT*^ mice. After 12 weeks of HFD feeding, tamoxifen was administered to induce *Dhhc5* deletion. In fasting and refeeding experiments, *Dhhc5-LECKO* mice exhibited a significantly slower increase in postprandial plasma triglycerides (Fig. 4a). Similarly, oral gavage of olive oil revealed impaired intestinal lipid absorption in *Dhhc5-LECKO* mice (Fig. 4b). Oil Red O staining of intestinal tissues further corroborated these findings (Fig. 4c). Together, these results demonstrate that DHHC5 regulates intestinal lipid uptake through its function in LECs.

### DHHC5 is required for VEGFR2 signaling in LECs

**Figure 5:**
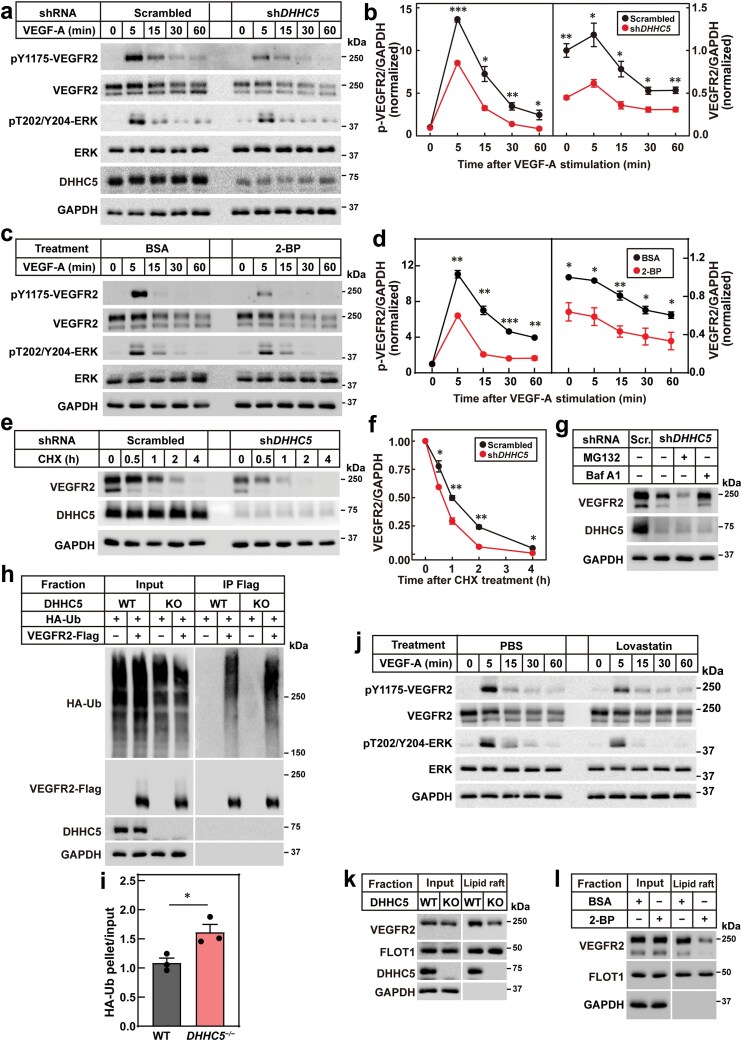
DHHC5 is required for VEGFR2 signaling in LECs. (a and b) Effects of VEGF-A treatment on VEGFR2 signaling detected by western blot analysis. Control (scrambled) and *DHHC5*-knockdown (sh*DHHC5*) HLECs were starved with FBS-free ECM for 6 h and then stimulated with 50 ng/mL VEGF-A at indicated time. Cells were harvested for western blot (a). The experiment was repeated three times independently, and the band intensities of VEGFR2 and pY1175-VEGFR2 were quantified and plotted in (b). Each value represents mean ± SEM. (c and d) Effects of 2-BP on VEGF-A-induced VEGFR2 signaling. HLECs were starved with FBS-free ECM for 6 h and meanwhile treated with 10% BSA or 100 μmol/L 2-BP. Then cells were stimulated with 50 ng/mL VEGF-A at indicated time and harvested for western blot analysis (c). The experiment was repeated three times independently, and the band intensities of VEGFR2 and pY1175-VEGFR2 were quantified and plotted in (d). Each value represents mean ± SEM. (e and f) Quantification of VEGFR2 protein treated with chlorhexidine (CHX) in the control and *DHHC5*-knockdown HLECs. Control and *DHHC5*-knockdown HLECs were treated with 50 mg/mL CHX for indicated time and then harvested for western blot analysis (e). The experiment was repeated three times independently, and the band intensities of VEGFR2 were quantified and plotted in (f). Each value represents mean ± SEM. (g) Quantification of VEGFR2 protein treated with MG132 or bafilomycin A1 in *DHHC5*-knockdown HLECs. Control and *DHHC5*-knockdown HLECs were cultured to confluence, and *DHHC5*-knockdown HLECs were treated with 10 μmol/L MG132 or 1 μmol/L bafilomycin A1 for 4 h. Cells were harvested for western blot analysis. (h and i) VEGFR2 ubiquitination analysis. On Day 0, wild type (WT) and *DHHC5*^*−/−*^ HEK293T cells were set up at 1.2 × 10^5^ cells per 35-mm dish. On Day 2, cells were transfected with 0.75 μg *Vegfr2*-FLAG/pcDNA3.3 and 0.75 μg Hemagglutinin (HA)-6X Ubiquitin/pCDH-puro plasmids. On Day 3, cells were treated with 1 μmol/L bafilomycin A1 for 4 h. Then cells were harvested and subjected to a double-IP protocol to detect ubiquitination of VEGFR2 (h). The experiment was repeated three times independently, and the band intensities of pellet HA-Ub were quantified and plotted in (i). Each value represents mean ± SEM. (j) Effects of lipid raft disruption on VEGF-A-induced phosphorylation of VEGFR2 and ERK. HLECs were starved with FBS-free ECM for 6 h and meanwhile treated with 10 μmol/L lovastatin. Then cells were stimulated with 50 ng/mL VEGF-A at indicated time and harvested for western blot analysis. (k) Effects of *DHHC5* knockdown on lipid raft association of VEGFR2. WT and *DHHC5*^*−/−*^ HEK293T cells were set up and transfected with 1.5 μg *Vegfr2*-FLAG/pcDNA3.3 plasmids. Cells were harvested and subjected to a lipid raft isolation protocol. (l) Effects of 2-BP on lipid raft association of VEGFR2. HEK293T cells were set up and transfected with 1.5 μg *Vegfr2*-FLAG/pcDNA3.3 plasmids. Before harvest, cells were treated with 10% BSA or 100 μmol/L 2-BP for 6 h and then subjected to a lipid raft isolation protocol. Asterisks (*) denote the level of statistical significance (Student’s *t*-test) between scramble and sh*DHHC5* HLECs. *P < 0.05; **P < 0.01; ***P < 0.001.

To elucidate how DHHC5 regulates the intestinal lymphatic system, we focused on VEGFR2, a key regulator of LEC homeostasis [12]. We knocked down *DHHC5* in human lymphatic endothelial cells (HLECs) and assessed VEGFR2 signaling. VEGF-A treatment in *DHHC5*-knockdown cells resulted in reduced phosphorylation of VEGFR2 and extracellular signal-regulated kinase (ERK), indicating impaired VEGFR2 signaling (Fig. 5a and b). Similar effects were observed with VEGF-C and VEGF-D treatment (Supplementary Fig. S5a–d).

To verify the results and explore whether the palmitoyl acyltransferase activity of DHHC5 is essential for its activity, we treated HLECs with 2-bromopalmitate (2-BP), a pan-DHHC inhibitor. Consistent with the knockdown results, 2-BP treatment markedly suppressed VEGF-A-induced VEGFR2 signaling (Fig. 5c and d).

We next investigated the mechanism by which DHHC5 regulates VEGFR2 signaling. While *VEGFR2* mRNA levels remained unchanged, VEGFR2 protein levels were significantly reduced in *DHHC5*-knockdown cells (Fig. 5a and b; Supplementary Fig. S5a–e). Further analysis revealed that DHHC5 deficiency accelerated VEGFR2 degradation, which was rescued by the lysosomal inhibitor bafilomycin A1 (Fig. 5e–g). Additionally, *DHHC5* knockout increased VEGFR2 ubiquitination (Fig. 5h and i).

To explore how DHHC5 stabilizes VEGFR2, we considered the role of protein palmitoylation in subcellular localization [27]. Surface biotinylation assays showed that *DHHC5* knockdown reduced the total surface content of VEGFR2, but did not alter the proportion of VEGFR2 localized to the plasma membrane (Supplementary Fig. S5f and g). This suggests that DHHC5 regulates VEGFR2 distribution rather than its surface localization.

Previous studies have shown that lipid raft association stabilizes VEGFR2 in endothelial cells [28]. We therefore examined whether DHHC5 is required for the lipid association of VEGFR2 in LECs. Disruption of lipid rafts using lovastatin, a cholesterol synthesis inhibitor, suppressed VEGF-A-induced phosphorylation of VEGFR2 and ERK (Fig. 5j). Furthermore, knockdown of *DHHC5* or treatment with 2-BP significantly reduced the lipid raft association of VEGFR2 (Fig. 5k and l; Supplementary Fig. S5h). These findings demonstrate that DHHC5 is essential for the lipid raft localization of VEGFR2, thereby stabilizing VEGFR2 and enabling its signaling.

### DHHC5 palmitoylates CRYBG1 to regulate the VEGFR2 signaling

We then hypothesized that DHHC5 might directly palmitoylate VEGFR2 or its co-receptor neuropilin 1 (NRP1) to regulate the lipid raft association of VEGFR2. However, resin-assisted capture of S-acylated proteins (Acyl-RAC) analysis revealed that VEGFR2 was not palmitoylated (Supplementary Fig. S6a). Although NRP1 was palmitoylated, it was not the substrate of DHHC5 (Supplementary Fig. S6b). Therefore, DHHC5 might regulate the lipid raft association of VEGFR2 through alternative substrate(s).

**Figure 6:**
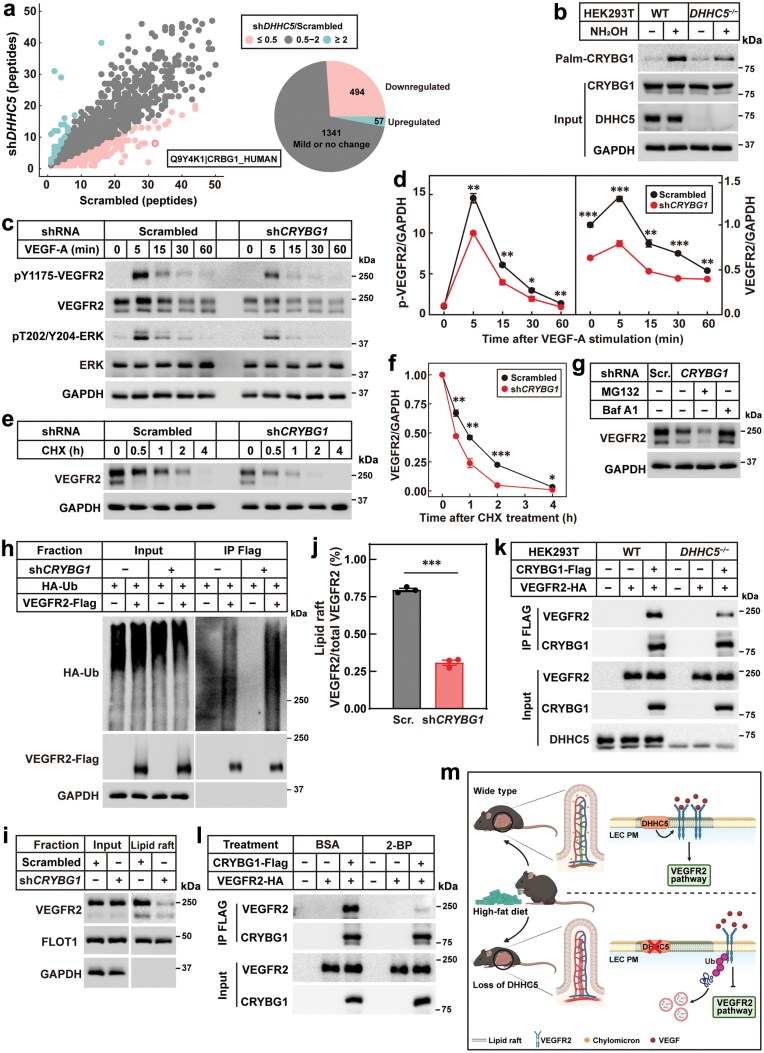
DHHC5 palmitoylates CRYBG1 to control the VEGFR2 signaling. (a) Mass spectrometry analysis of palmitoylated proteins from control and *DHHC5*-knockdown HLECs. Control and *DHHC5*-knockdown HLECs were subjected to Acyl-RAC assay. The eluted fractions were subjected to mass spectrometry for protein ID analysis. Each dot represented one protein identified. X and Y axes represent the number of unique peptides of each protein in the two groups. (b) CRYBG1 palmitoylation in WT and *DHHC5*^*−/−*^ cells. WT and *DHHC5*^*−/−*^ HEK293T cells were set up and transfected with 1.5 μg *Crybg1*-FLAG/pCDH-puro plasmids. Cell lysates were subjected to Acyl-RAC assay. (c and d) Effects of *CRYBG1* knockdown on VEGF-A-induced VEGFR2 signaling. Control (scrambled) and *CRYBG1*-knockdown (sh*CRYBG1*) HLECs were starved with FBS-free ECM for 6 h and then stimulated with 50 ng/mL VEGF-A at indicated time. Cells were harvested for western blot analysis (c). The experiment was repeated three times independently, and the band intensities of VEGFR2 and pY1175-VEGFR2 were quantified and plotted in (d). Each value represents mean ± SEM. (e and f) Effects of *CRYBG1* knockdown on VEGFR2 stability. Control and *CRYBG1*-knockdown HLECs were treated with 50 mg/mL CHX for indicated time and then harvested for western blot analysis (e). The experiment was repeated three times independently, and the band intensities of VEGFR2 were quantified and plotted in (f). Each value represents mean ± SEM. (g) Effects of *CRYBG1* deficiency on VEGFR2 degradation. Control and *CRYBG1*-knockdown HLECs were cultured to confluence and *CRYBG1*-knockdown HLECs were treated with 10 μmol/L MG132 or 1 μmol/L bafilomycin A1 for 4 h. Cells were harvested for western blot. (h) Effects of *CRYBG1* deficiency on VEGFR2 ubiquitination. Scrambled and *CRYBG1*-knockdown HEK293T cells were set up and transfected with plasmids as in Fig. 5g. Before harvest, cells were treated with 1 μmol/L bafilomycin A1 for 4 h. Then cells were harvested and subjected to a double-IP protocol to detect ubiquitination of VEGFR2. (i and j) Effects of *CRYBG1* deficiency on lipid raft content of VEGFR2. Scrambled and *CRYBG1*-knockdown HEK293T cells were set up and transfected with 1.5 μg *Vegfr2*-FLAG/pcDNA3.3 plasmids. Cells were harvested and subjected to a lipid raft isolation protocol (i). The experiment was repeated three times independently, and the band intensities of lipid raft and total VEGFR2 were quantified and plotted in (j). Each value represents mean ± SEM. (k) Effects of *DHHC5* knockout on the interaction between CRYBG1 and VEGFR2. WT and *DHHC5*^*−/−*^ HEK293T cells were set up and transfected with 0.75 μg *Crybg1*-FLAG/pCDH-puro and 0.75 μg *Vegfr2*-HA/pcDNA3.3 plasmids. Cell lysates were subjected to a co-IP protocol to detect the interaction between CRYBG1 and VEGFR2. (l) Effects of 2-BP treatment on the interaction between CRYBG1 and VEGFR2. HEK293T cells were set up and transfected with plasmids as in (k). Before harvest, cells were treated with 10% BSA or 100 μmol/L 2-BP for 6 h. Then cell lysates were subjected to a co-IP protocol to detect the interaction between CRYBG1 and VEGFR2. (m) A schematic illustration of the role of DHHC5 and CRYBG1 in controlling lacteal function and intestinal lipid absorption by maintaining the lipid raft association of VEGFR2. Asterisks (*) denote the level of statistical significance (Student’s *t*-test) between scramble and sh*CRYBG1* HLECs. *P < 0.05; **P < 0.01; ***P < 0.001.

To identify the substrate(s), we isolated palmitoylated proteins from control and *DHHC5*-knockdown HLECs and subjected them to mass spectrometry analysis. Among the 1892 detected proteins, 494 proteins showed at least a 2-fold decrease in *DHHC5*-knockdown cells (Fig. 6a). CRYBG1, an actin-binding protein implicated in cancer progression [29–32] but with no reported role in LECs, emerged as a candidate. First, we confirmed that *DHHC5* knockdown reduced CRYBG1 palmitoylation (Fig. 6b). Second, *CRYBG1* knockdown significantly impaired VEGF-A-induced VEGFR2 signaling (Fig. 6c and d; Supplementary Fig. S6c) and decreased VEGFR2 stability (Fig. 6e and f). Third, CRYBG1 deficiency promoted VEGFR2 ubiquitination and lysosomal degradation (Fig. 6g and h) and dramatically reduced the lipid raft content of VEGFR2 (Fig. 6i and j). Together, these findings demonstrate that CRYBG1 is a DHHC5 substrate essential for regulating VEGFR2 signaling.

To further elucidate how CRYBG1 maintains the lipid raft localization of VEGFR2, we performed co-immunoprecipitation (co-IP) assays. CRYBG1 interacted with VEGFR2, and this interaction was weakened by *DHHC5* knockout (Fig. 6k and Supplementary Fig. S6d–f) or 2-BP treatment (Fig. 6l), indicating that CRYBG1 palmitoylation is required for its binding to VEGFR2.

## Discussion

Although the intestinal lymphatic system is known to play a critical role in lipid absorption [12, 15, 33, 34], the regulatory mechanisms underlying its function remain poorly understood. We demonstrate that the palmitoyl acyltransferase DHHC5 is essential for intestinal lipid absorption by maintaining the integrity of the intestinal lymphatic system. DHHC5 sustains the VEGFR2 signaling in LECs by promoting the lipid raft localization of VEGFR2. Loss of DHHC5 disrupts the intestinal lymphatic system and inhibits intestinal lipid absorption (Fig. 6m). These findings highlight the regulatory role of protein palmitoylation in LEC homeostasis and provide new insights into the molecular mechanisms governing intestinal lipid metabolism.

Our study also uncovers a regulatory mechanism of VEGFR2. Rather than directly palmitoylating VEGFR2, DHHC5 palmitoylates CRYBG1, an actin-binding protein that interacts with VEGFR2 and is required for its lipid raft association. CRYBG1 has previously been implicated in cytoskeletal regulation, inhibiting cytoskeletal remodeling in prostate epithelial cells [29]. Given that cytoskeletal contraction is essential for chylomicron uptake by LECs [15], CRYBG1 might regulate the lacteal function by modulating both chylomicron uptake and lipid raft localization of VEGFR2. Notably, flotillin-2, another DHHC5 substrate [35], regulates cytoskeletal dynamics and membrane trafficking [36]. While we did not investigate flotillin-2 in this study, its potential role in DHHC5-mediated lipid absorption cannot be ruled out and warrants further exploration.

Notably, the *Dhhc5-LECKO* mice only partially recapitulated the lipid absorption defects seen in *Dhhc5-IKO* mice, suggesting that DHHC5 may also function in other tissues to regulate lipid absorption. Our findings that DHHC5 depletion in intestinal epithelial cells, hepatocytes, or pancreas had no significant effect on lipid absorption indicate that these tissues are not the primary sites of DHHC5 action. Future studies should aim to identify additional tissues or cell types through which DHHC5 regulates lipid absorption.

While palmitoylation typically directs cargo proteins into certain membrane microdomains [18], CRYBG1 remains in the lipid rafts even in the absence of DHHC5. This suggests that CRYBG1 may utilize alternative anchoring mechanisms, such as intrinsic lipid-binding domains [37] or interaction with VEGFR2 via its transmembrane domain. Indeed, palmitoylation has been shown to regulate protein–protein interactions without altering subcellular localization [38]. In this study, palmitoylation primarily facilitates the binding of CRYBG1 to VEGFR2 rather than its lipid raft localization.

### Limitations of the study

This study has several limitations. First, while we demonstrate that lacteals are blocked and dysfunctional in *Dhhc5-IKO* mice, technical challenges prevented us from directly assessing changes in lacteal cell junctions. We hypothesize that loss of DHHC5 disrupts lacteal junctions, impairing chylomicron uptake and leading to inflammation. Second, inducible knockout of *Dhhc5* in LECs (*Dhhc5-LECKO* mice) caused transient defects in lipid absorption and body weight loss, which resolved after 2 weeks. This may be due to the fact that *Prox1*-Cre^ERT^ targets mature LECs [39] but not LEC stem cells, allowing for the replacement of DHHC5-deficient LECs. However, the lack of a suitable antibody for DHHC5 immunostaining in LECs prevented us from confirming this hypothesis. Future studies using improved antibodies or Cre drivers targeting LEC stem cells could address this question.

## Materials and methods

### Mice

*Dhhc5* ^*flox/flox*^ (*Dhhc5*^*f/f*^) mice were generated and *Pdx1-Cre* mice were purchased from Shanghai Research Center for Model Organism. *Rosa26-Cre*^*ERT*^ mice were purchased from Jackson Laboratory. *Prox1-Cre*^*ERT*^ mice [39] were a generous gift from Dr. Jin Li at Fudan University. *Villin-Cre* and *Albumin-Cre* mice were generous gifts from Dr. Sheng-Cai Lin at Xiamen University. All mice were housed in colony cages at 22°C with 12-h light/12-h dark cycles. The dark cycle began at 7 p.m. All animal studies were performed with the approval of the Institutional Animal Care and Research Advisory Committee at Xiamen University and Fudan University. All mice used in this study were male on a C57BL/6 background.

The chow diet (Xietong Organism, Nanjing, China) contains 12% calories from fat, 67.4% from carbohydrates, and 20.6% from protein. The HFD (Research Diet, D12492) contains 60% calories from fat, 20% calories from carbohydrate, and 20% calories from protein.

### Plasmids

Full-length cDNAs of mouse *Vegfr2*, *Crybg1*, and *Nrp1* were cloned from a cDNA library prepared from the testis of a C57BL/6 mouse. The coding regions of these genes were cloned into either pcDNA3.3 or pCDH-EF1-MCS-IRES-Puro (System Biosciences) with C-terminal HA or Flag tags.

For knockdown, shRNAs were designed and cloned into pLKO.1 (Addgene, 10878). The primer sequences are listed in Supplementary Table S1.

### Cell culture

HEK293T cells were cultured in high-glucose Dulbecco’s modified Eagle’s medium (DMEM, Thermo Fisher Scientific) supplemented with 10% (v/v) fetal calf serum (FCS, VivaCell), 100 U/mL penicillin, and 100 mg/mL streptomycin at 37°C in an atmosphere of 5% CO_2_. HLECs, purchased from Guangzhou Jennio Biotech Co., Ltd, were cultured in ECM (endothelial cell medium containing FCS, endotheliao cell growth supplement (ECGS), and penicillin/streptomycin (P/S), ScienCell, Catalog No. 1001) at 37°C in an atmosphere of 5% CO_2_. *DHHC5*^*−/−*^ HEK293T cells were previously generated in our laboratory [20].

### Lentivirus production and infection

Lentivirus was produced in HEK293T cells using psPAX2 (Addgene, 12260) and pMD2.G (Addgene, 12259) as the packaging plasmids [20]. Medium containing lentiviral particles was either concentrated at 70,000 *g* for 2 h or directly aliquoted and stored at −80°C until use. For infection of HLECs and HEK293T cells, the cells were cultured to about 70% confluence and infected with lentivirus in medium containing 10 μg/mL polybrene. After 24 h, the cells were selected against 5 μg/mL puromycin for at least 48 h before further use.

### Palmitoylated protein analysis by Acyl-RAC assay

Palmitoylated proteins were isolated and detected by Acyl-RAC assay as previously described [20]. Briefly, 0.1% S-methyl methanethiosulfonate (S-MMTS) was used to block free thiol groups at 42°C for 15 min. Then proteins were precipitated by cold acetone at −20°C for 1 h, washed twice by cold 70% acetone, and then resuspended in 300 μL Buffer A (100 mmol/L hydroxyethylpiperazine ethane sulfonic acid (HEPES), pH 7.5, 1 mmol/L ethylenediaminetetraacetic acid (EDTA), and 1% odium dodecyl sulfate (SDS)). A 60 μL volume of the sample was mixed with loading buffer as input. A 120 μL volume of the sample was mixed with 10 μL thiopropyl sepharose 6B and 40 μL 2 mol/L NH_2_OH. The other 120 μL of the sample was mixed with an equal volume of thiopropyl sepharose 6B and 2 mol/L NaCl as a negative control. Samples were rotated at room temperature (RT) for at least 3 h. Beads were washed with Buffer A containing 8 mol/L urea for five times (5 min each) and eluted with 60 μL Buffer A containing 50 mmol/L dithiothreitol (DTT) at RT for 20 min. Eluted fractions were mixed with loading buffer and analyzed by mass spectrometry or western blot.

### Surface biotinylation

Surface biotinylation was carried out as previously described [40] with some modifications [20]. Briefly, HLECs were washed with cold phosphate-buffered saline (PBS) and incubated with freshly prepared sulfo-NHS-SS-biotin (0.5 mg/mL) in PBS for 30 min on ice. Cells were then washed three times with ice-cold quenching buffer (PBS containing 50 mmol/L glycine, pH 7.4). Cells were lysed in PBS containing 1% Triton X-100, and supernatant was incubated with streptavidin agarose at 4°C for 2 h. Beads were washed five times with PBS containing 1% Triton X-100, and eluted with SDS loading buffer at 37°C for 1 h.

### Immunoprecipitation and western blot

HEK293T cells were lysed in radioimmunoprecipitation assay (RIPA) buffer (50 mmol/L Tris, pH 7.4, 150 mmol/L NaCl, 1% Triton X-100, 0.1% SDS, 1 mmol/L EDTA, and 1 mmol/L ethylene glycol tetraacetic acid (EGTA)) containing protease and phosphatase inhibitors (ApexBio). Cell lysate was subjected to immunoprecipitation using anti-Flag M2 beads. Pellet was washed five times with wash buffer (50 mmol/L Tris, pH 7.4, 150 mmol/L NaCl, 0.1% Triton X-100, and 0.1% SDS), and incubated with SDS loading buffer at 37°C for 1 h.

The following antibodies were used for western blot: anti-Flag (Sigma-Aldrich, F1804, 1:1000), anti-HA (Proteintech, 51064-2-AP, 1:1000), anti-VEGFR2 (Proteintech, 26415-1-AP, 1:1000), anti-pY1175 VEGFR2 (Abclonal, AP0382, 1:1000), anti-ERK (Proteintech, 16443-1-AP, 1:1000), anti-Phospho-p44/42 MAPK (CST, 4377S, 1:1000), anti-DHHC5 (Sigma-Aldrich, HPA014670, 1:1000), anti-ATP1A1 (Abcam, ab167390, 1:1000), anti-Flotillin-1 (BD Biosciences, 610820, 1:1000), anti-Calnexin (CST, 2679s, 1:1000), and anti-GAPDH (Proteintech, 60004-1-Ig, 1:10,000). Blots were developed using MiniChemi910 (SINSAGE) and band intensities were quantified using ImageJ.

### Ubiquitination assay

A double immunoprecipitation assay to detect the ubiquitination of VEGFR2 was performed as previously described [41, 42]. Briefly, *DHHC5*-WT/KO HEK293T cells were co-transfected with VEGFR2-FLAG and HA-6X ubiquitin. Before harvest, cells were treated with 1 μmol/L bafilomycin A1 for 6 h. Cells were lysed in RIPA buffer and subjected to immunoprecipitation using anti-Flag M2 beads at 4°C for 4 h. After washing with wash buffer for five times, beads were incubated with 0.1 mL Buffer B (50 mmol/L Tris, 150 mmol/L NaCl, pH 7.4, and 1% SDS) at 37°C for 1 h. The eluted fractions were diluted 10 times with RIPA buffer and subjected to another round of immunoprecipitation using anti-Flag M2 beads. After washing with wash buffer for five times, beads were incubated with SDS loading buffer at 37°C for 1 h and subjected to western blot using anti-HA antibody.

### Isolation of lipid rafts

Cells were washed with ice-cold PBS and lysed in hypotonic buffer (20 mmol/L HEPES, pH 7.4, 10 mmol/L KCl, 1.5 mmol/L MgCl_2_, and 1 mmol/L EDTA) containing protease/phosphatase inhibitors. Mechanical disruption was achieved by 22-gauge needle shearing. Complete lysis was verified microscopically. A portion of lysate was reserved as input control (mixed with SDS buffer and sonicated). Remaining lysate was centrifuged (1000 *g*, 10 min, 4°C) to remove nuclei. Membrane fractions were pelleted by high-speed centrifugation (20,000 *g*, 30 min, 4°C). Membrane pellets were treated with 1% Triton X-100 in PBS for 1 h at 4°C. After centrifugation (20,000 *g*, 30 min), detergent-insoluble lipid rafts were solubilized in SDS buffer with sonication. All samples were heated at 37°C before immunoblotting analysis.

### Immunofluorescence

For whole-mount immunostaining of lacteals, the jejunum was dissected, rinsed with PBS, and fixed with 4% formaldehyde for 4 h at 4°C. After washing with PBS, the jejunum was embedded in optimal cutting temperature (OCT) medium. Cryosections with 100-µm thickness were cut in a freezing microtome. The cryosections were rinsed with PBS to remove OCT medium completely and preserve the sheet-like jejunum only. The jejunum was blocked with a buffer containing 3% bovine serum albumin (BSA) and 0.3% Triton-X 100 for 1 h at RT. The jejunum was incubated with primary antibodies (diluted in blocking buffer with 3-fold dilution) for 2 days at 4°C, washed with PBS containing 0.1% Triton X-100 (3 × 45 min) and incubated with fluorescent labeled-secondary antibodies overnight at 4°C. Following washing with PBST for three times (45 min each), the jejunum was stained with 4',6-Diamidino-2-Phenylindole (DAPI) for 10 min at RT and then washed with PBST. The jejunum was mounted in a fluorescent mounting medium (DAKO Inc.) Images were taken using a Zeiss LSM-880 confocal microscopy in a 3D Z-stack mode. Anti-LYVE-1 (AngioBio, 11-034; 1:500) and anti-F4/80 (BioLegend, 123119, 1:100) antibodies were used.

### Metabolic cage analysis

Metabolic cage analysis was performed in a home-cage system Phenomaster (TSE Systems) at 22°C with a 12-h light/12-h dark cycle. Mice were acclimated to the metabolic cages for 2 days after being housed singly for a week. Mice were monitored for at least 4 days on changes in body weight, amount of food intake, levels of O_2_ consumption, rates of CO_2_ production, and extents of locomotor activities.

### Observation of chylomicrons by TEM

Mice were fasted for 16 h and then refed with HFD for 2 h. Mice were sacrificed, and blood samples were collected (with 500 mmol/L EDTA to prevent blood clotting) and then centrifuged at 3500 rpm for 5 min at 4°C. Plasma was collected and centrifuged again at 50,000 rpm for 5 h at 4°C. Chylomicrons were in the milky liquid on the upper layer. Chylomicrons were collected and negatively stained immediately, and then observed by a transmission electron microscope Hitachi HT-7800.

### Histology

At the end of the feeding, mice were dissected and tissue sections of inguinal subcutaneous white adipose tissue (iWAT), gonadal white adipose tissue (gWAT), brown adipose tissue (BAT), liver, and small intestine (duodenum and jejunum) were fixed for 2 days in 10% (v/v) paraformaldehyde in PBS. The fixed tissues were embedded in paraffin and sectioned at 5 μm. Slides were stained with hematoxylin and eosin (H&E), and imaged as previously described [43, 44].

### Oral glucose tolerance test

Oral glucose tolerance test was performed as previously described [45]. Briefly, mice were fasted for 16 h (from 5 p.m. to 9 a.m.) and gavaged with D-glucose at 2 mg/g body weight for chow diet-fed mice and 1 mg/g body weight for HFD-fed mice. Blood samples were collected from tail vein at 0, 15, 30, 60, 90, and 120 min after gavage, and blood glucose was measured by a Bayer Contour Glucometer.

### Flow cytometry analysis

Blood samples were collected from mouse orbit and lysed twice by 1 mL ACK lysing buffer to remove red blood cells. Leukocytes were washed and resuspended in fluorescence-activated cell sorting (FACS) buffer, and then stained with antibodies. BV510-CD4 (BioLegend, 563106, 1:400), BV421-CD8a (BioLegend, 100737, 1:400), FITC-Ly-6G (BioLegend, 561105, 1:400), PE-Cy7-CD3e (eBioscience, 25-0038-41, 1:400), APC-B220 (BioLegend, 103212, 1:400), and APC-Cy7-Zombie dye (BioLegend, 77184, 1:400) antibodies were used.

### Quantitative real-time PCR

Total RNA was isolated, and quantitative real-time PCR (qRT-PCR) measurements were performed as described [46], using the SYBR master mix (Yeasen, Shanghai). The primers are listed in Supplementary Table S1. The relative amount of each mRNA was calculated by using the comparative threshold cycle (CT) method. Cyclophilin or 36B4 was used as the invariant control.

### Quantification and statistical analysis

All the statistical analysis was performed using Student’s two-tailed paired *t*-test. The value represents mean ± SEM. Statistical details of all experiments can be found in the figure legends, including the exact number of cell samples or mice. Asterisks (*) indicate levels of statistical significance. *P < 0.05; **P < 0.01; ***P < 0.001. No data were excluded from any of the experiments.

## Supplementary Material

loaf014_suppl_Supplementary_Materials

## Data Availability

The authors confirm that all the data supporting the findings of this study are available within the supplementary material and corresponding authors.
