# Supplementary material for: DHHC5 regulates lacteal function and intestinal lipid absorption by maintaining VEGFR2 localization in lipid rafts
Source: Life Metab. 2025 Apr 10;4(4):loaf014. doi: 10.1093/lifemeta/loaf014 (PMC12207882; doi:10.1093/lifemeta/loaf014)
Supplement: loaf014_suppl_Supplementary_Materials [file loaf014_suppl_supplementary_materials.pdf]

Supplementary Information

DHHC5 regulates lacteal function and intestinal lipid absorption by maintaining VEGFR2 localization in lipid rafts

Zhao et al.

Supplementary Figure S1

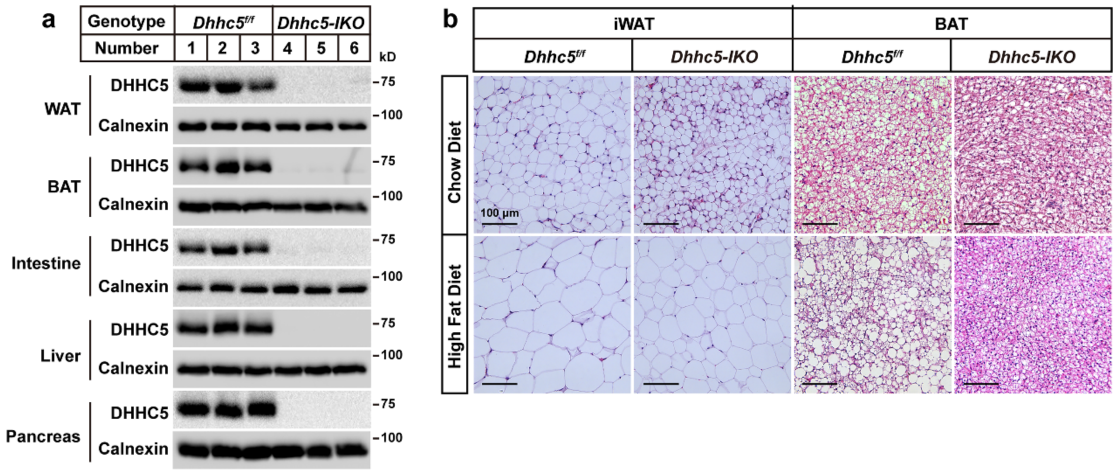

Supplementary Figure S1 Knockout of *Dhhc5* in adult mice protects against diet-induced obesity. (a) WAT, BAT, intestine, liver, and pancreas of *Dhhc5*$^{f/f}$ and *Dhhc5-IKO* mice were collected and subjected to western blot to analyze the knockout efficiency of *Dhhc5*. (b) iWAT and BAT of *Dhhc5*$^{f/f}$ and *Dhhc5-IKO* mice were collected from the experiments in Figure 1a and subjected to H&E analysis. Scale bar, 100 μm.

Supplementary Figure S2

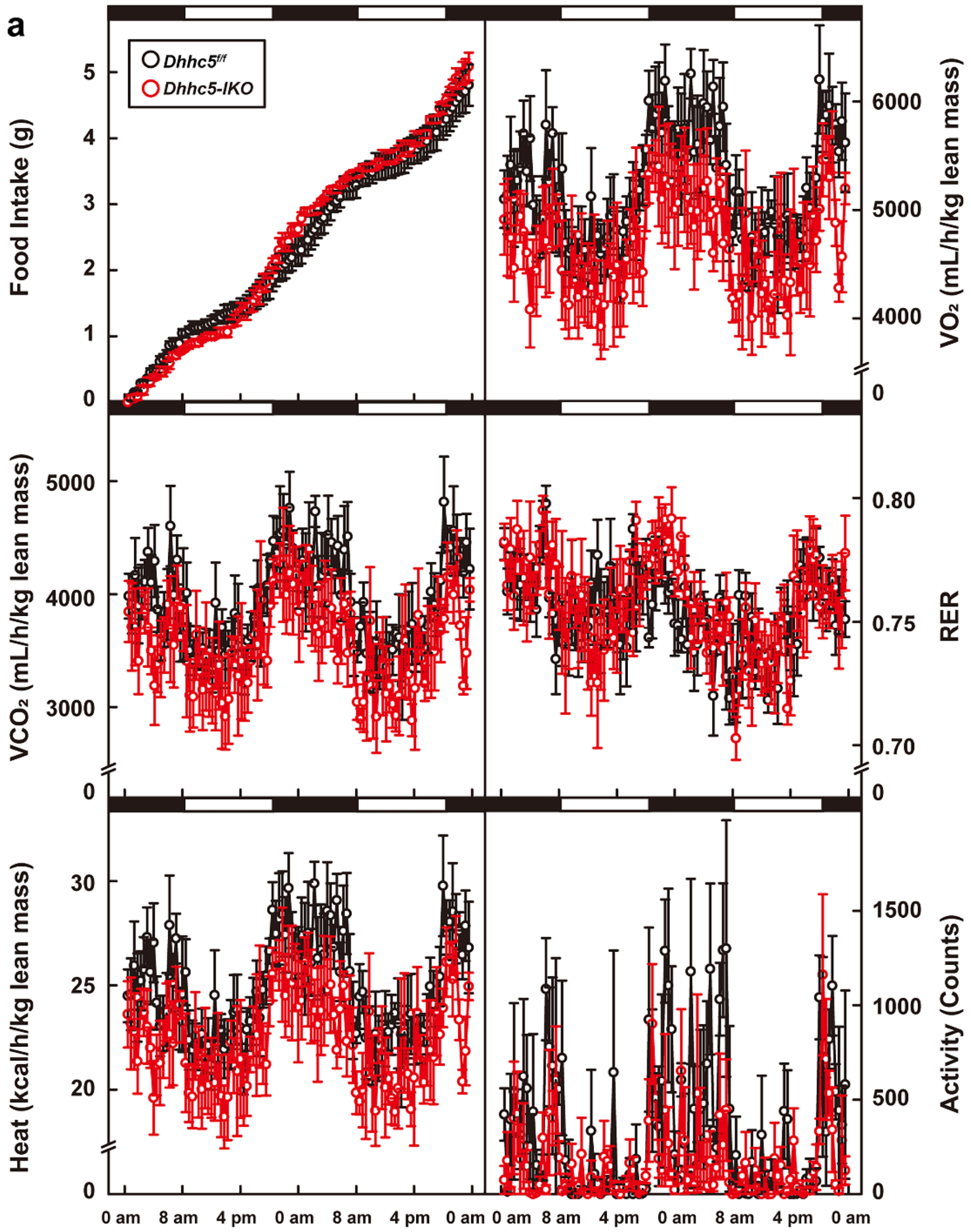

Supplementary Figure S2 *Dhhc5-IKO* mice do not show differences in metabolic cage analysis. HFD-fed *Dhhc5*$^{f/f}$ and *Dhhc5-IKO* mice (16-week-old, male) were subjected to metabolic cage analysis. Food intake, O_2 consumption, CO_2 production, respiratory exchange ratio (RER), heat production, and activity in 2 consecutive days are presented. Each value represents mean ± SEM of 4 mice.

Supplementary Figure S3

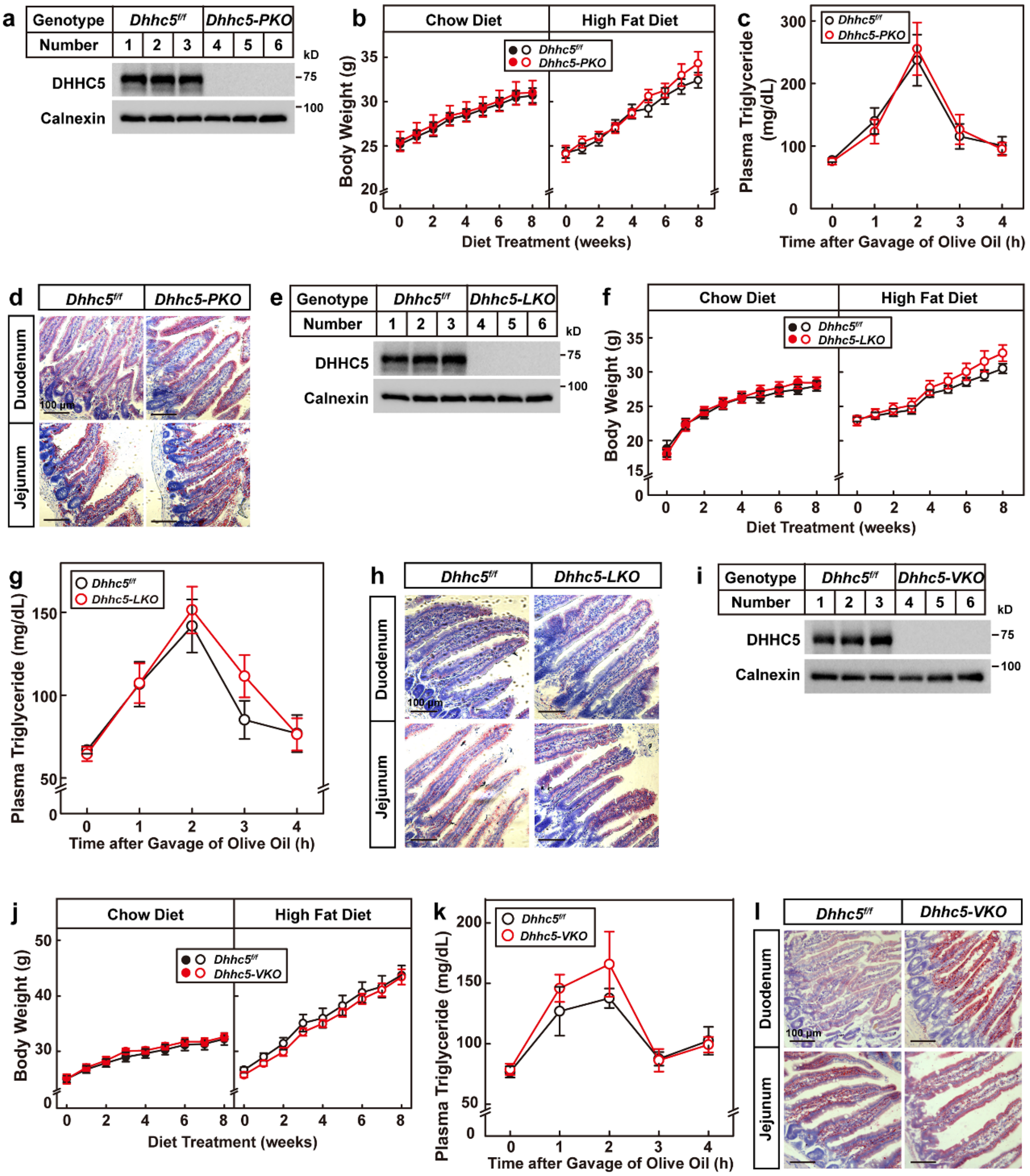

Supplementary Figure S3 *Dhhc5-VKO*, *Dhhc5-LKO*, and *Dhhc5-PKO* mice do not have defects in intestinal lipid absorption. At week 8, *Dhhc5$^{f/f}$* and *Dhhc5-PKO* mice (a−d), *Dhhc5$^{f/f}$* and *Dhhc5-LKO* mice (e−h), and *Dhhc5$^{f/f}$* and *Dhhc5-VKO* mice (i−l) were subjected to chow diet or HFD feeding for 8 weeks, respectively. (a, e, and i) Pancreas of *Dhhc5$^{f/f}$* and *Dhhc5-PKO* mice (a), liver of *Dhhc5$^{f/f}$* and *Dhhc5-LKO* mice (e), and enterocytes of *Dhhc5$^{f/f}$* and

Dhhc5-VKO mice (i) were isolated and subjected to western blot to verify the knockout efficiency of *Dhhc5*. (b, f, and j) Body weight was monitored every week. Each value represents mean ± SEM of 9 mice in (b) and (f), mean ± SEM of 8 chow diet-fed mice or 5 HFD-fed mice in (j). (c, g, and k) HFD-fed mice (16-week-old, male) were subjected to lipid absorption analysis as in Figure 2a. Each value represents mean ± SEM of 6 mice in (c) and (g), mean ± SEM of 5 mice in (k). (d, h, and l) HFD-fed mice (17-week-old, male) were subjected to Oil Red O staining as shown in Figure 2c. Scale bar, 100 μm.

Supplementary Figure S4

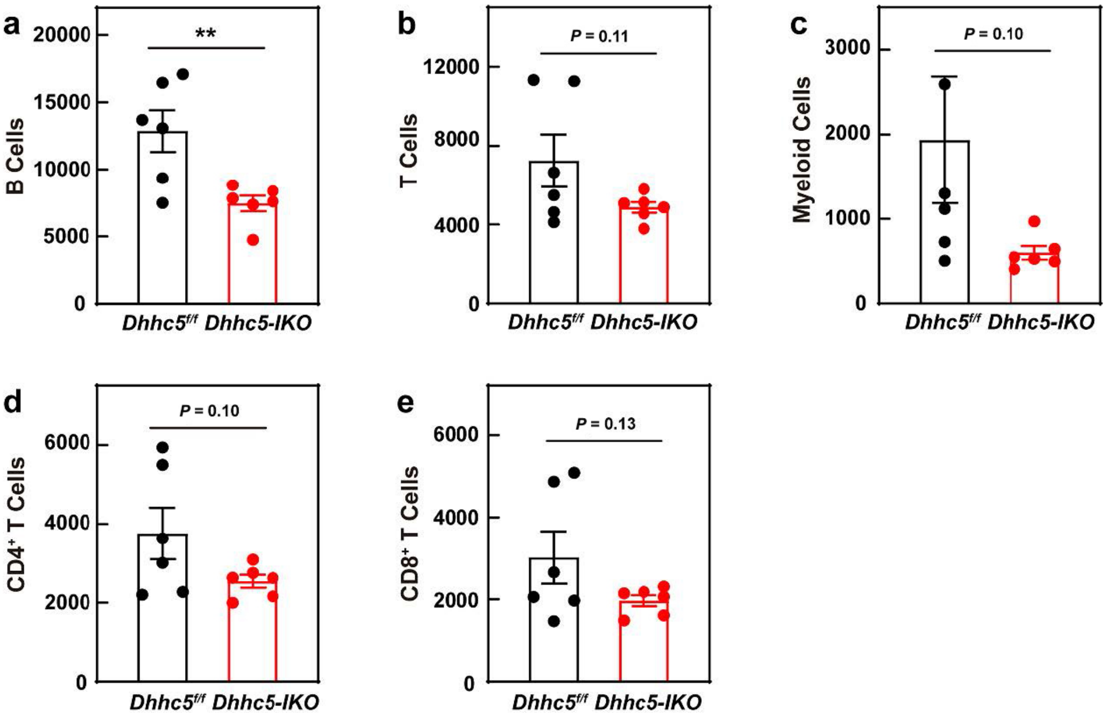

Supplementary Figure S4 HFD-fed *Dhhc5-IKO* mice have decreased blood immune cells. Blood samples of *Dhhc5$^{f/f}$* and *Dhhc5-IKO* mice were collected from the experiments in Figure 3e and subjected to flow cytometry analysis of blood immune cells. (a and b) Cells were analyzed by the marker B220. B and T cells were analyzed by the marker CD3. (c) Myeloid cells were analyzed by the marker Ly6G. (d and e) T cells were subdivided into CD4$^+$ and CD8$^+$ T cells by the marker CD4 and CD8, respectively. Each value represents mean ± SEM of 6 mice. Asterisks (*) denote the level of statistical significance (Student's *t*-test) between *Dhhc5$^{f/f}$* and *Dhhc5-IKO* mice. $^{**}P < 0.01$.

Supplementary Figure S5

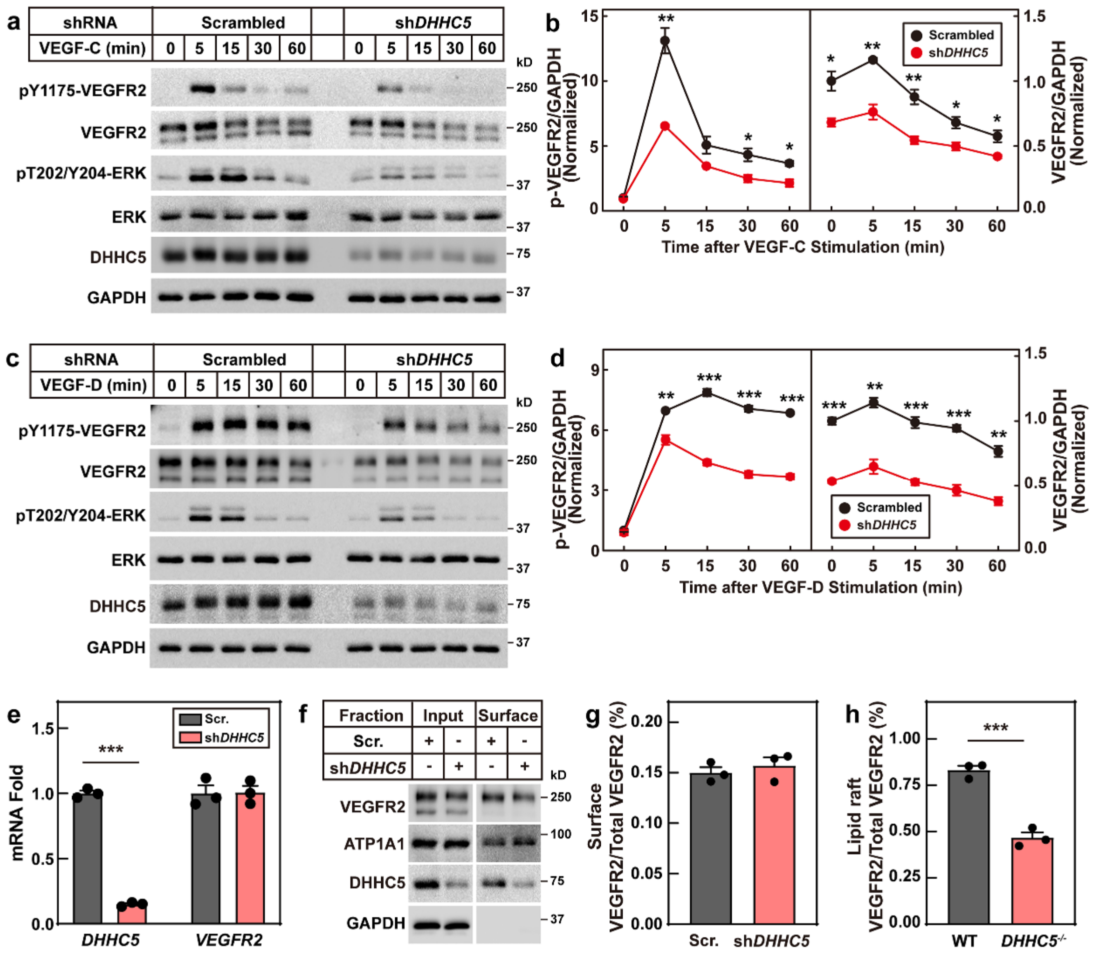

Supplementary Figure S5 DHHC5 is required for VEGFR2 signaling in LECs. (a−d) Control and *DHHC5*-knockdown HLECs were starved with FBS-free ECM for 6 h and then stimulated with 100 ng/mL VEGF-C (a and b) or 200 ng/mL VEGF-D (c and d) at indicated time. Cells were harvested for western blot analysis (a and c). The experiment was repeated three times independently and the band intensities of VEGFR2 and pY1175-VEGFR2 were quantified and plotted in (b and d). Each value represents mean ± SEM. (e) Control and *DHHC5*-knockdown HLECs were harvested and total RNA was isolated. The mRNA level of *VEGFR2* was detected by RT-PCR. Each value represents mean ± SEM of three replicates. (f and g) Control and *DHHC5*-knockdown HLECs were starved with FBS-free ECM for 6 h and then

subjected to a surface biotinylation protocol (f). The experiment was repeated three times independently and the band intensities of total and surface VEGFR2 were quantified and plotted in (g). Each value represents mean ± SEM. (h) The experiment in Figure 5i was repeated three times independently and the band intensities of total and lipid raft VEGFR2 were quantified and plotted. Each value represents mean ± SEM. Asterisks (*) denote the level of statistical significance (Student's *t*-test) between scrambled and sh*DHHC5* HLECs. $^{*}P < 0.05$; $^{**}P < 0.01$; $^{***}P < 0.001$.

Supplementary Figure S6

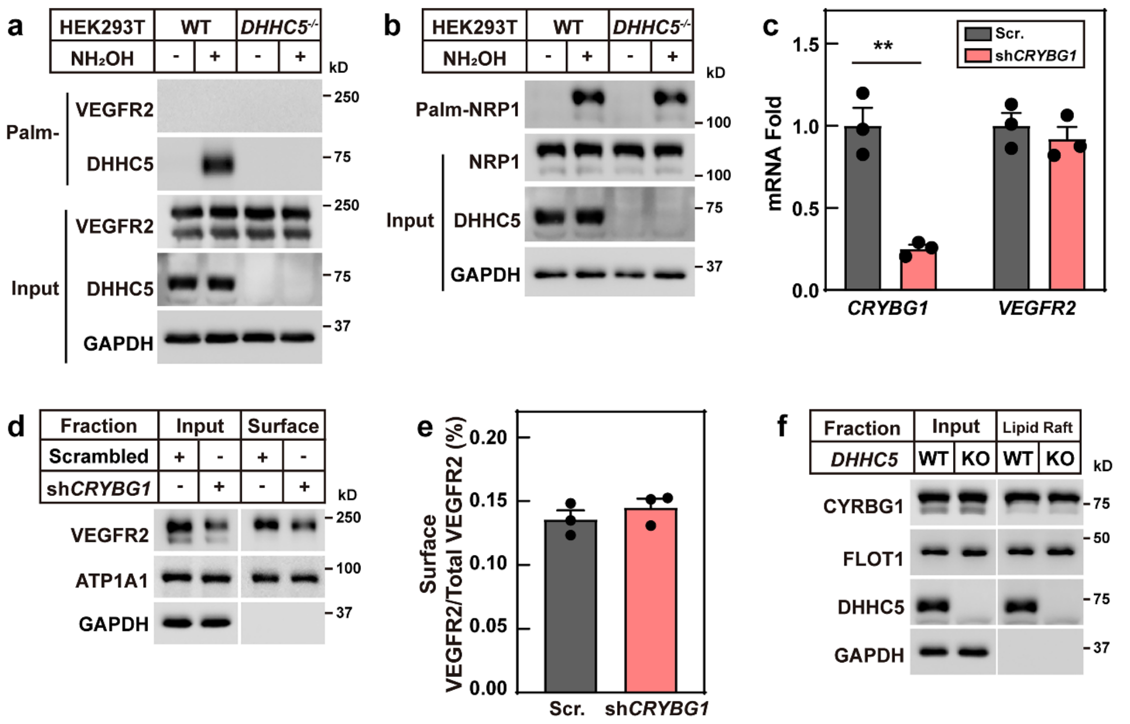

Supplementary Figure S6 DHHC5 palmitoylates CRYBG1 to control the VEGFR2 signaling. (a and b) WT and *DHHC5*$^{-/-}$ HEK293T cells were set up and transfected with 1.5 μg *Vegfr2*-FLAG/pcDNA3.3 (a) or 1.5 μg *Nrp1*-FLAG/ pcDNA3.3 (b) plasmids. Cell lysates were subjected to Acyl-RAC assay. (c) Control and *CRYBG1*-knockdown HLECs were harvested and total RNA was isolated. The mRNA levels of *CRYBG1* and *VEGFR2* were detected by RT-PCR. Each value represents mean ± SEM of three replicates. (d and e) Control and *CRYBG1*-knockdown HLECs were starved with FBS-free ECM for 6 h and then subjected to a surface biotinylation protocol (d). The experiment was repeated three times independently and the band intensities of total and surface VEGFR2 were quantified and plotted in (e). Each value represents mean ± SEM. (f) WT and *DHHC5*$^{-/-}$ HEK293T cells were set up

and transfected with 1.5 µg *Crybg1*-FLAG/pcDNA3.3 plasmids. Cells were harvested and subjected to a lipid raft isolation protocol.

Supplementary Table S1 Primer information.

Primers	Source of primer sequences
**(a) Primers to generate different constructs**	
h*DHHC5*-shRNA	TRCN0000166569 (Mission shRNA, Sigma-Aldrich)
h*CRYBG1*-shRNA	TRCN0000160150 (Mission shRNA, Sigma-Aldrich)
**(b) Quantitative real-time PCR primers**	
h*DHHC5*-Forward	5'-GTTTGGCTTTGGCCTCCTTTA-3'
h*DHHC5*-Reverse	5'-ACACACATTACTGCCATTGTGAC-3'
h*VEGFR2*-Forward	5'-GGCCCAATAATCAGAGTGGCA-3'
h*VEGFR2*-Reverse	5'-CCAGTGTCATTTCCGATCACTTT-3'
h*CRYBG1*-Forward	5'-CCTCCTATTCACGAAGACCA-3'
h*CRYBG1*-Reverse	5'-AGGCTCACTGTTCCCATTAG-3'
